# Prostaglandin E1 Is an Efficient Molecular Tool for Forest Leech Blood Sucking

**DOI:** 10.3389/fvets.2020.615915

**Published:** 2021-01-07

**Authors:** Fenshuang Zheng, Min Zhang, Xingwei Yang, Feilong Wu, Gan Wang, Xingxing Feng, Rose Ombati, Ruiling Zuo, Canju Yang, Jun Liu, Ren Lai, Xiaodong Luo, Chengbo Long

**Affiliations:** ^1^Department of Emergency Medicine, Second People's Hospital of Yunnan Province, Kunming, China; ^2^Key Laboratory of Animal Models and Human Disease Mechanisms of Chinese Academy of Sciences & Yunnan Province, Kunming Institute of Zoology, Kunming, China; ^3^Kunming College of Life Science, University of Chinese Academy of Sciences, Kunming, China; ^4^State Key Laboratory of Phytochemistry and Plant Resources in West China, Kunming Institute of Botany, Chinese Academy of Sciences, Kunming, China; ^5^Department of Clinical Laboratory, Kunming Children's Hospital, Kunming, China; ^6^Dali People's Hospital of Yunnan Province, Dali, China; ^7^Dehong People's Hospital, Mangshi, China; ^8^Sino-African Joint Research Center, Chinese Academy of Sciences, Wuhan, China

**Keywords:** leech, blood-sucking, prostaglandin E1, anticoagulant, *Haemadipsa sylvestris*

## Abstract

From a survival perspective, it is hypothesized that leech saliva exhibits certain physiological effects to ensure fast blood-feeding, including analgesia, anesthesia, and anti-inflammation to stay undetected by the host and vasodilatation and anti-hemostasis to ensure a steady, rapid, and sustained blood flow to the feeding site. Many anti-hemostatic compounds have been identified in leech saliva, such as hirudin, calin, and bdellin A. However, no specific substance with direct vasodilatory and anti-inflammatory function has been reported from forest leech saliva. Herein, using activity-guided analysis, prostaglandin E1 (PGE1) was identified for the first time as an efficient molecular tool for forest leech blood sucking. The structure of PGE1 was analyzed by nuclear magnetic resonance spectroscopy and high-resolution electrospray ionization mass spectroscopy. PGE1 was found to be primarily distributed in the leech salivary gland (1228.36 ng/g body weight). We also analyzed how forest leech PGE1 affects platelet aggregation, skin vascular permeability, bleeding time, and pain. Results indicated that PGE1 efficiently inhibited platelet aggregation induced by adenosine diphosphate (ADP) (5 μM) with an IC_50_ of 21.81 ± 2.24 nM. At doses of 10, 100 nM, and 1 μM, PGE1 increased vascular permeability by 1.18, 5.8, and 9.2 times. It also prolonged bleeding time in a concentration-independent manner. In the formalin-induced mouse paw pain model, PGE1 suppressed acute pain. To the best of our knowledge, this is the first report on PGE1 in invertebrates. The functions of PGE1, such as vasodilation, platelet aggregation inhibition, anti-inflammation, and pain alleviation, may facilitate the ingestion of host blood by leeches.

## Introduction

Animal venom facilitates survival by antagonizing prey and predators (1). As exclusively blood-sucking ectoparasites, leeches must penetrate the body surface of their host and suppress host reactions to injury, swelling, pain, and inflammation in order to remain undetected and successfully obtain a blood meal. Leeches counteract these reactions by injecting saliva into their hosts. This suggests that analgesic or anesthetic agents may be delivered to the host during the blood-sucking process. Furthermore, leeches must overcome hemostatic and vasoconstriction reactions in the host to ensure a steady and sustained blood flow to the feeding site (2, 3).

Blood-sucking leeches have been used for medical purposes in humans for more than two thousand years and are approved as medicinal agents in many countries (4). For example, the Food and Drug Administration of USA (FDA) approved the use of leeches in plastic and reconstructive surgery in 2004 (5) and leech therapy was recently authorized as a legal therapeutic intervention in Europe. Increasing attention has also been paid to medicinal leech therapy in pain syndromes (6) as its analgesic effects appear to be rapid, effective, and long-lasting in various conditions (7).

Many components containing anticoagulant and antithrombotic functions have been identified from leech saliva. Leeches can ingest a large amount of blood (>10-fold their own body weight) from their hosts within a short duration (20–30 min) (8), suggesting that they sustain host vasodilation and peripheral perfusion increase to take-up large volumes of blood while keeping the feeding time as short as possible (2). We previously revealed an analgesic peptide from *Haemadipsa sylvestris* (3). However, the specific analgesic, vasodilatory, and anti-inflammatory substances in leech saliva, and their corresponding pharmacological mechanisms, are yet to be identified.

PGE1 (alprostadil) belongs to the eicosanoid family. As an agonist of PGE1 receptors, the compound has extensive physiological and pharmacological effects, including platelet aggregation inhibition, vasodilation, and inflammation response modulation. Therefore, it is often used for erectile dysfunction and vascular diseases (9). Here, we identified PGE1 as the main component of platelet aggregation inhibition in the salivary gland of *H. sylvestris*. Previously only found in mammals, this is the first report of PGE1 in an invertebrate. Its presence in saliva may help leeches successfully obtain blood meals.

## Materials and Methods

### Collection of Saliva Samples From *H. sylvestris*

*Haemadipsa sylvestris* (total weight 16 kg) leeches were collected from a forest in Chuxiong, Yunnan Province, China. The live leeches were quickly frozen in liquid nitrogen and stored at −80°C. All experimental protocols using these animals were approved by the Animal Care and Use Committee at the Kunming Institute of Zoology, Chinese Academy of Sciences (SYDW-2013018).

### Purification of Platelet Aggregation-Inhibiting Components

**Figure 1:**
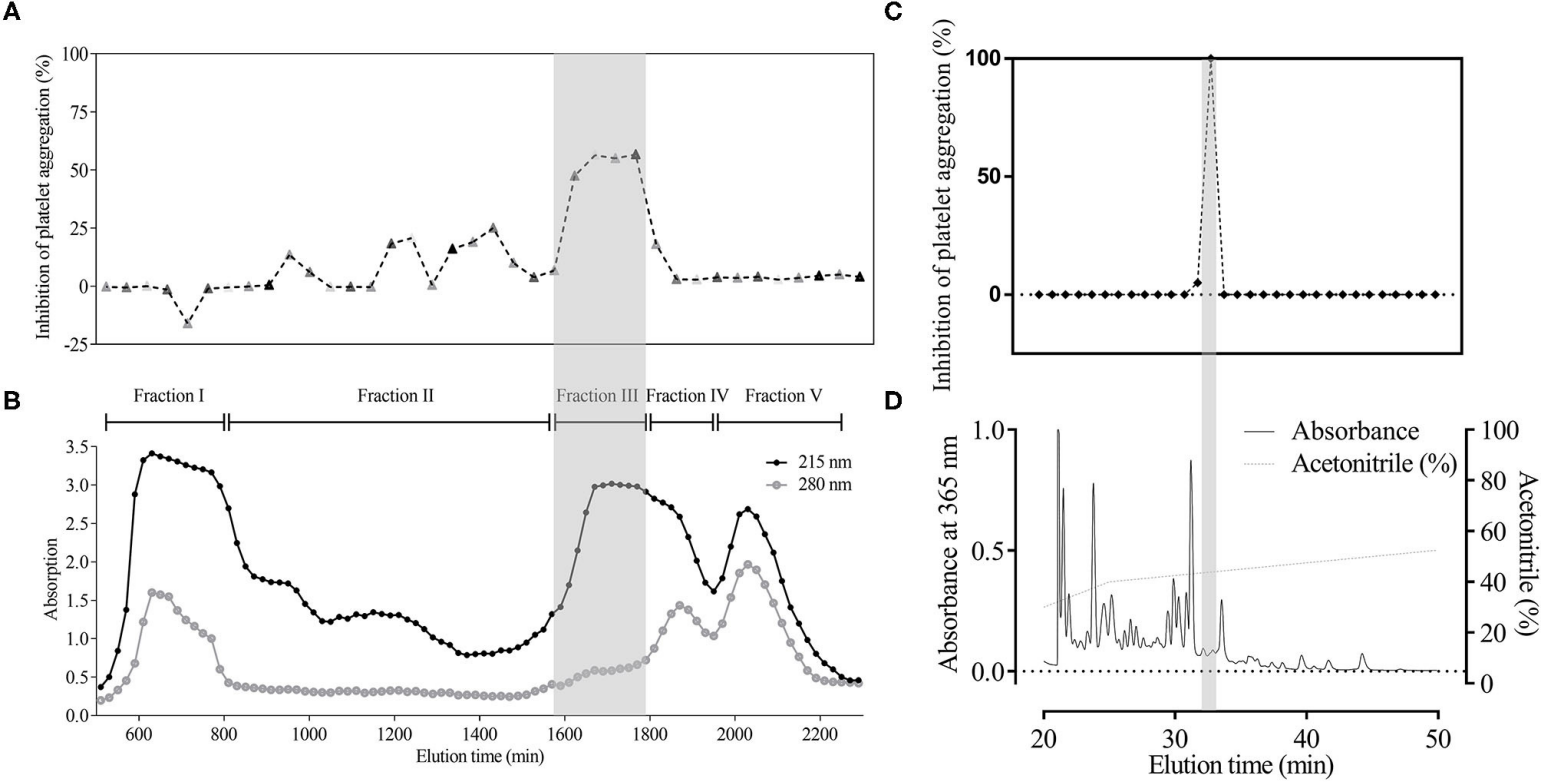
Major component with platelet aggregation-inhibiting activity in *H. sylvestris*. Using activity-guided analysis, crude extract from the heads (500 mg) was divided into five fractions by G-50 gel filtration column according to 280 and 215 nm absorption. G-50 gel **(B)** and bioassay data **(A)** were obtained simultaneously. Fraction III (gray shadow) accounted for 60% of inhibited platelet aggregation. Fraction III was collected and further analyzed by C_18_ reverse-phase (RP) column. Substance absorption was monitored at 215 nm. Samples were gathered every minute, and bioactivity was tested immediately. Results showed that PGE1 was the only active compound (gray shadow) **(C,D)**.

Using activity-guided purification, the platelet aggregation-inhibiting components were purified. Briefly, 15 kg of leeches was extracted by 95% ethanol at 4°C for 24 h. The ethanol extract was separated by solvent extraction with petroleum ether and ethyl acetate. The active fraction (ethyl acetate phase) was further purified by silica gel column chromatography, gel exclusion chromatography (Sephadex LH20), and high-performance liquid chromatography (HPLC, C18). The purification and HPLC procedures are shown in Figures 1A,B, respectively.

### Structural Analysis

High-resolution electrospray ionization mass spectroscopy (HRESIMS) was recorded on a Waters Auto Premier P776 (Waters, MA, USA). One-dimensional (1-D) and 2-D nuclear magnetic resonance (NMR) spectra were detected on a Bruker DRX-800M Hz spectrometer (Bruker, MA, USA) with trimethylsilane as an internal standard. Chemical shifts (δ) were expressed in ppm with reference to standard signals. Optical rotations were obtained with a Horiba SEPA-300 polarimeter (Horiba Ltd., Kyoto, Japan).

### Distribution of PGE1 in *H. sylvestris*

*Haemadipsa sylvestris* (total weight 500 g) leeches were collected from the outdoors in Yunnan Province, China. The live leeches were quickly frozen in liquid nitrogen, followed by the dissection and collection of their heads (1/4 of body length), coeloms (middle), and posterior suckers. Each part was homogenized in Tri-HCl buffer (50 mM, pH 8.0), and centrifuged at 12,000 × *g* for 10 min at 4°C to remove debris. The PGE1 concentration in the resulting supernatant was determined using an enzyme-linked immunosorbent assay (ELISA) kit (ab133044, Abcam, Cambridge, MA, USA) following the manufacturer's instructions.

To investigate the contribution of PGE1 to total platelet aggregation inhibition activity, crude extract of the head samples was applied to a Sephadex G-50 gel filtration column (GE Healthcare, 2.6 × 100 cm) equilibrated with 50 mm Tris-HCl buffer (pH 8.9). Sample fractionation was performed by eluting the column using the Tri-HCl buffer. Each eluted fraction (3.0 ml) was collected after 10 min and absorbance was measured at 280 nm and 215 nm. The fractions that exhibited platelet aggregation inhibition activity were pooled and analyzed using reverse-phase HPLC (SunFire™ Prep C18, 250 × 10 mm, Waters, Milford, MA, USA) with a acetonitrile gradient elution system containing 0.1% trifluoroacetic acid (TFA) on a Waters 1525 pump with a detector (Waters 2489). The platelet aggregation-inhibiting activities of the collected fractions were tested and gathered every minute. Samples showing activity were analyzed by liquid chromatography-tandem mass spectrometry (LC-MS/MS) (3).

### Assays on Platelet Aggregation Inhibition

Fresh blood obtained from healthy donors was centrifuged at 175 × *g* for 10 min and 3,000 × *g* for 10 min at room temperature (25°C) to acquire platelet-rich plasma (PRP) and platelet-poor plasma (PPP), respectively. Finally, the platelet pellet was resuspended at a density of 3 × 10^5^ platelets/μl in PPP. Aliquots of PRP (280 μl) were incubated with 10 μl of sample (dissolved in 0.9% saline) for 5 min at 37°C. Platelet aggregation induced by 10 μl of ADP (final concentration of 5 μM) was monitored by light transmission in an aggregometer (Plisen, Beijing, China) with continuous stirring at the same temperature. The inhibition of platelet aggregation was measured as the maximum aggregation response within 300 s.

### Skin Vascular Permeability Assay

Vascular permeability was analyzed with a Miles assay (10). Briefly, anesthetized (2.5% pentobarbital sodium, 100 μl) female Kunming mice (18–22 g) were injected with 0.25% Evans Blue solution (10 μl/g body weight) through the caudal vein. After 5 min, 40 μl of 0.9% saline (negative control), histamine (positive control, 100 μM, dissolved in 0.9% saline), or PGE1 (dissolved in 0.9% saline) was injected subcutaneously into the dorsal skin of the mice. After 30 min, the animals were sacrificed, and their skins were removed to allow the injection sites to be photographed. Evans Blue dye in the skin samples was extracted by incubation with formamide overnight at 55°C. After centrifugation at 20,000 × *g* for 60 min at 25°C, the Evans Blue concentration in the supernatant was determined by measuring absorbance at 620 nm.

### Bleeding Time

After anesthesia with pentobarbital sodium, mice were placed into the prone position. Different concentrations of PGE1 solution and 100 μM histamine solution (all dissolved in saline) were injected intravenously. After 10 min, a 10-mm tail-tip was transected with a scalpel. The tail was immediately immerged in a 100-mL flask containing 50 ml of saline solution heated to 37°C during the experiment. Each mouse was monitored for 30 min. The saline solution of each mouse was mixed well, with absorbance tested at 540 nm to evaluate blood loss (11).

### Analgesic Effect

The analgesic effects of PGE1 were tested using the formalin-induced paw pain model. Histamine and formalin (Sigma Aldrich, USA) were used as positive controls. Briefly, the mice (18–20 g) were placed in a transparent observation chamber (40 × 20 × 20 cm). After 30 min of adaptation, 2% formalin solution, different concentrations of PGE1 solution, and 100 μM histamine solution (all dissolved in saline) were injected subcutaneously into the pad of the left hind paw at a volume of 20 μl. The mice were returned to the chamber immediately. Paw licking behavior of the mice was recorded with a timer over 45 min. The early nociceptive response phase (phase I) and late phase (phase II) were 0–5 min and 15–45 min, respectively (12).

### Data Analysis

Data are given as mean ± standard error of the mean (SEM). Statistical analyses were performed using two-tailed Student's *t*-test. *P* ≤ 0.05 were considered significant.

## Results

### Identification of Main Platelet Aggregation-Inhibiting Component From *H. sylvestris*

Using activity-guided analysis, crude extract of the head samples (500 mg) was analyzed using G-50 gel filtration column and HPLC with C18 reverse-phase (RP) column. We identified a major component showing platelet aggregation inhibition in the leech salivary gland, which accounted for ~60% of the activity (Figure 1). The HPLC active fraction was analyzed by LC-MS/MS and showed a molecular ion peak at m/z 353 [M-H]- under negative ion mode (**Figure 3A**).

**Figure 2:**
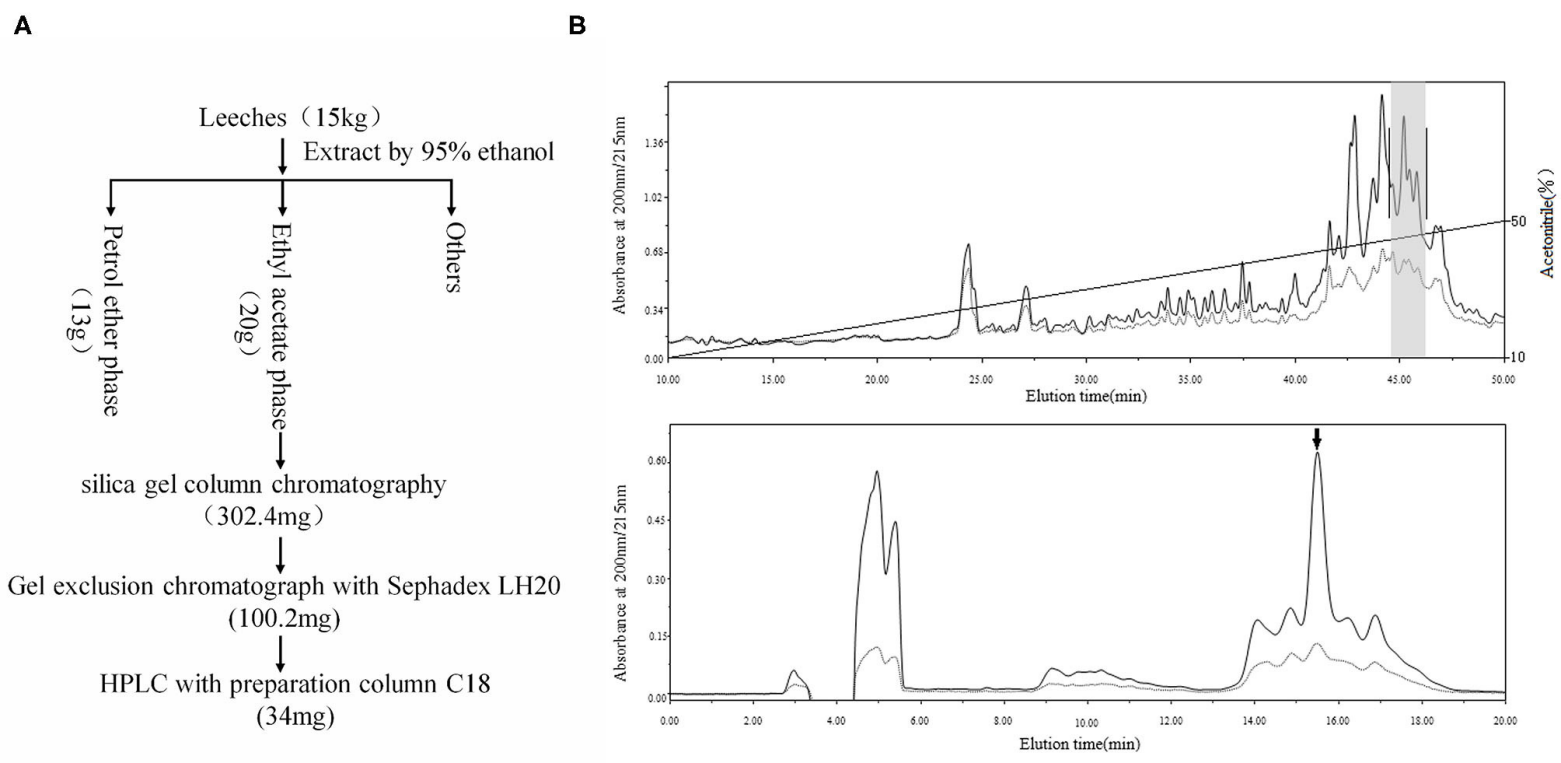
Purification of PGE_1_. **(A)** Using activity-guided purification, 15 kg of leeches was extracted by 95% ethanol at 4°C for 24 h. Ethanol extract was separated by solvent extraction with petroleum ether and ethyl acetate. Active fraction (20 g) was further purified by silica gel column chromatography, gel exclusion chromatography (Sephadex LH20), and HPLC (C_18_). Finally, we obtained 34 mg of sample for further purification. **(B)** The sample was further purified by C_18_ reverse-phase (RP) column using an acetonitrile gradient elution system containing 0.1% trifluoroacetic acid (TFA) (fraction with inhibitory effect on platelet aggregation is indicated by gray shadow, upper); active sample was purified again by C_18_ reverse-phase (RP) column using an isocratic elution model (42% acetonitrile) with the same solution, with PGE1 highlighted by an arrow (down).

The above results indicated that the active ingredient was likely a compound rather than a protein. Thus, we applied compound purification to obtain more of the sample. The extracts obtained from the 15 kg of leeches were separated. Only one compound was found to inhibit platelet aggregation effectively (Figure 2). Finally, we obtained 1 mg of active compound, as a colorless oil, which was then structurally analyzed.

**Figure 3:**
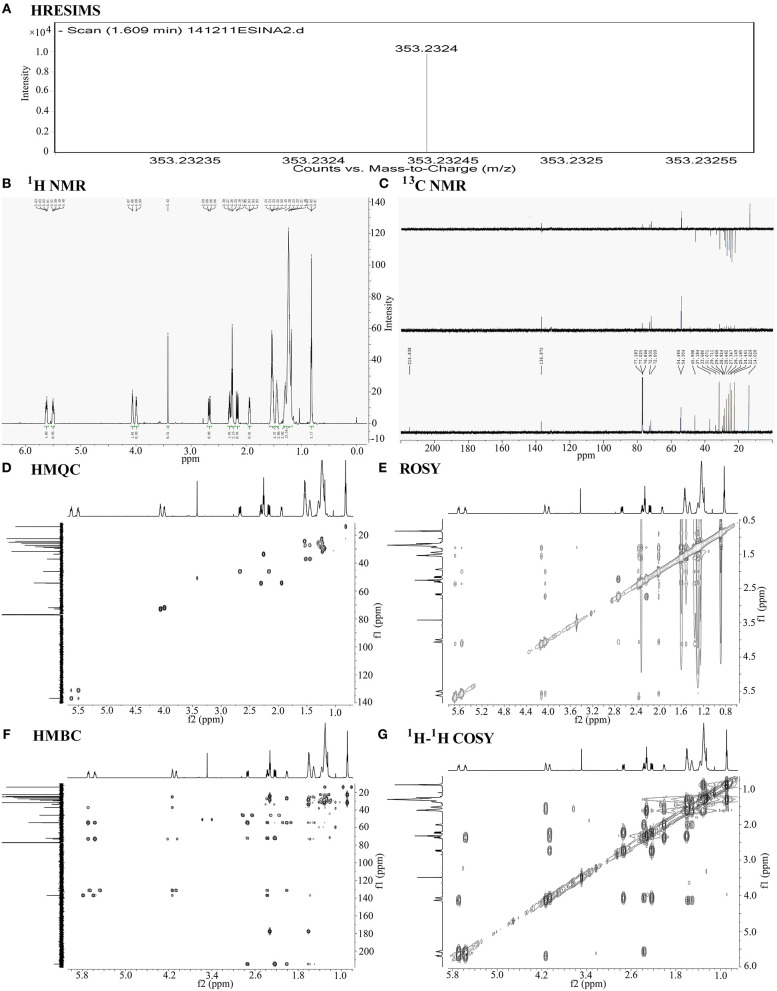
Chemical structural analysis of PGE1. **(A)** High-resolution electrospray ionization mass spectroscopy (HRESIMS) of PGE1. HRTOFMS m/z for C20H34O5 [M-H]- calculated 353.2328, found 353.2324. **(B)** 1H-NMR of PGE1 {chemical shift value: 5.70–5.65 (m, 1H), 5.57–5.53 (m, 1H), 4.13–4.09 (d, J = 6.0 Hz, 1H), 4.06–4.02 (d, J = 9.0 Hz, 1H), 2.74–2.69 (dd, J =18.0, 8.0 Hz, 1H), 2.37–2.33 (q, J = 10.0 Hz, 1H), 2.32–2.28 (t, J = 6.0 Hz, 2H), 2.24–2.18 (dd, J = 18.1, 8.0 Hz, 1H), 2.01–1.97 (m, 1H), 1.61–1.54 (m, 4H), 1.52–1.45 (m, 2H), 1.38–1.18 (m, 15H), 0.90–0.83 (m, 3H) and **(C)** 13C-NMR{chemical shift value: 214.8 (s), 177.8 (s), 136.9 (d), 131.2 (d), 72.9 (d), 72.0 (d), 54.5 (d), 54.3 (d), 46.0 (t), 37.2 (t), 33.7 (t), 31.7 (t), 28.9 (t), 28.5 (t), 27.3 (t), 26.1 (t), 25.2 (t), 24.4 (t), 22.6 (t), 14.0 (q).} of PGE1 in CDCl3. **(D–G)** 2-D NMR spectra (HMQC, HMBC, ROSY, and 1H−1H COSY) of PGE1 in CDCl3.

**Figure 4:**
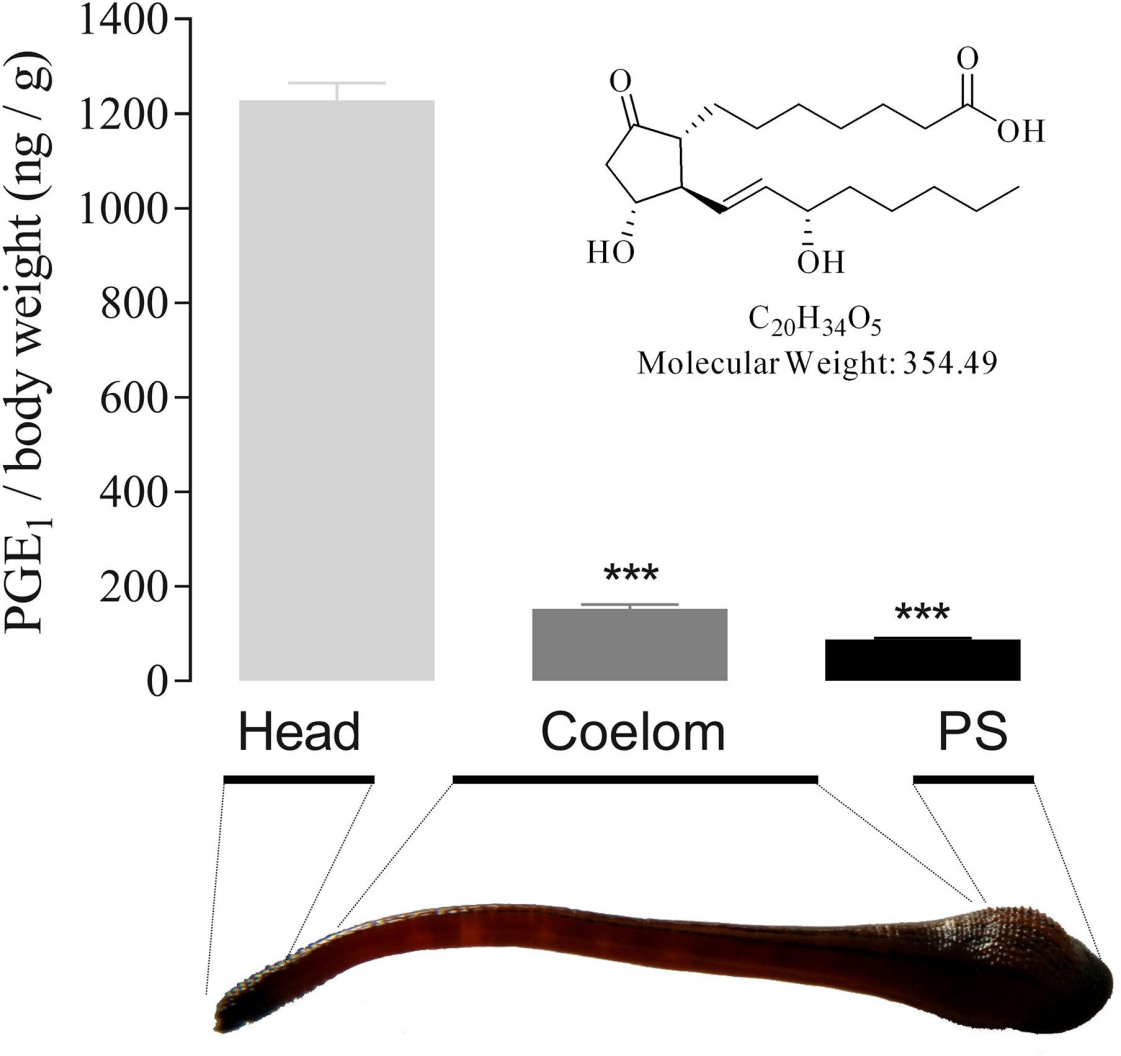
PGE_1_ structure and tissue distribution in *H. sylvestris*. Using activity-guided purification, 1 mg of active component was obtained from 15 kg of *H. sylvestris*. Structural analysis revealed the active substance to be PGE1. PGE1 tissue distribution in *H. sylvestris* was analyzed by ELISA. Heads showed the greatest quantity of PGE1 (1 228.36 ng/g body weight) compared with coelom and posterior sucker (PS). Statistically significant differences compared with heads of control group (two-tailed Student *t*-test) are indicated by ***P < 0.001.

The compound exhibited a molecular ion peak at m/z 353.2324 [M-H]- based on HRESIMS (Figure 3A). Together with the 13C NMR (Figure 3C) spectral data, the molecular formula was determined to be C20H34O5 with four degrees of unsaturation. The 13C NMR and DEPT (Distortionless Enhancement by Polarization Transfer) spectra displayed signals for one ketone, one carboxyl carbon, double bonds, four methines (two oxygenated), 11 methylenes, and one methyl, thus assigning one ring and a chain moiety to the structure. The 13C and 1H NMR (Figure 3B) spectral data were similar to those of prostaglandin, especially PGE1 (13–18). Based on detailed comparison of the MS and NMR spectral data and optical rotation with that of PGE1, we tentatively identified our compound as PGE1. The positions and configurations of the functional groups in the structure were further supported by detailed analysis of 1-D and 2-D NMR spectral data (Figures 3D–G). Thus, the main compound with platelet aggregation-inhibiting activity was determined to be PGE1 (Figure 4).

### PGE1 Distribution in Leech Tissues

To clarify its role in blood sucking, the tissue distribution of PGE1 in the leech body was analyzed. Results indicated that the highest quantity of PGE1 was detected in the head (1228.36 ng/g body weight), some 8 and 14 times higher than that found in the coelom (152.80 ng/g) and posterior sucker (88.18 ng/g), respectively (Figure 4). These findings suggest that PGE1 is mainly distributed in the leech salivary gland as a specific substance for inhibition of platelet aggregation.

### Functions of PGE1 Related to Blood Sucking

The PGE1 receptors, EP1-4 (prostaglandin E-type receptors) and IP (prostaglandin I-type receptors), are widely and variously distributed in the tissues and organs of animals. The differences in binding ability between the receptors and PGE1 allow it to perform different biological functions under various concentrations, which are, in turn, balanced by the receptors. PGE1 can bind to EP2, EP4, and IP to increase intracellular cAMP concentrations. In contrast, PGE1 can combine with EP1 and EP3 to increase intracellular Ca^2+^ and decrease intracellular cAMP concentrations, respectively (19, 20).

**Figure 5:**
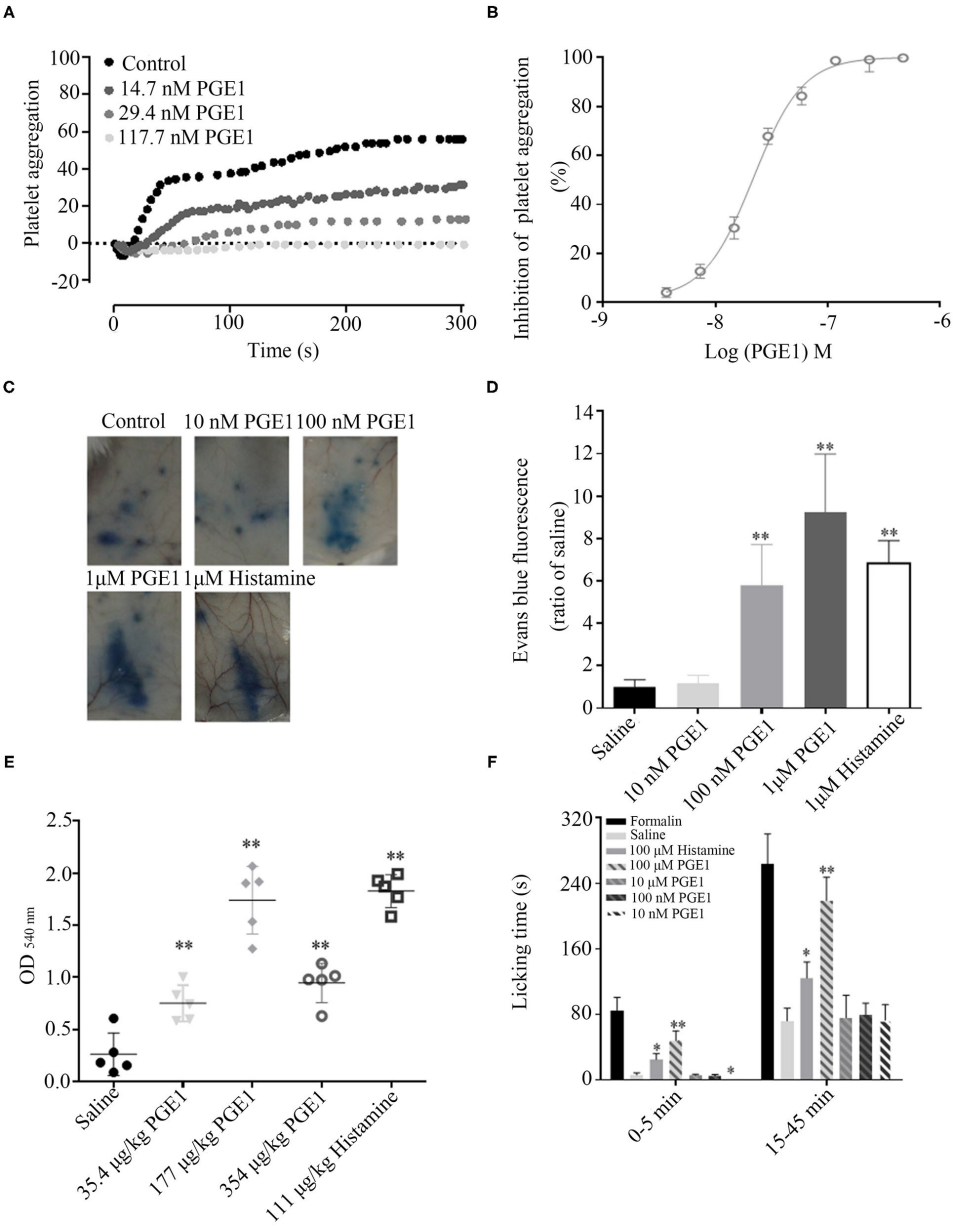
Functions of PGE1 to facilitate blood sucking. **(A,B)** PGE1 inhibited platelet aggregation in a dose-dependent manner with an IC_50_ of 21.81 nM *in vitro*. **(C,D)** PGE1 significantly promoted vascular permeability in a concentration-dependent manner *in vivo*. At doses of 10, 100 nM, and 1 μM, PGE1 increased vascular permeability by 1.18, 5.8, and 9.2 times, respectively, compared with the control (same volume of saline). **(E)** By intravenous injection of different concentrations of PGE1, bleeding time was prolonged in a concentration-independent manner. At doses of 35.4, 177, and 354 μg/kg, blood loss increased 2.7, 6.2, and 3.4 times, respectively, compared with normal control (same volume of saline). **(F)** In mouse paw pain model, PGE1 significantly suppressed acute pain at a dose of 10 nM. *P < 0.05 and **P < 0.01, significantly different to control.

Platelets mainly express EP 3–4 and IP receptors, and only a small amount of EP2. PGE1 plays an important role in inhibiting platelet activation by combining with IP and EP3, with IP playing a major role. PGE1 can effectively bind to IP receptors at low or high concentrations but can only bind to EP3 at high concentrations. Thus, PGE1 can effectively inhibit platelet aggregation at different concentrations (21). As illustrated in Figures 5A,B, forest leech PGE1 efficiently inhibited platelet aggregation induced by ADP (5 μM) with an IC_50_ of 21.81 ± 2.24 nM.

Additionally, PGE1 has vasodilatation and analgesic functions. Vasodilatation induces vascular permeability and extends bleeding time, ensuring that leeches can suck blood effectively. PGE1 significantly promoted vascular permeability in a concentration-dependent manner *in vivo*. Compared with the control (same volume of saline), at doses of 10, 100 nM, and 1 μM, PGE1 increased vascular permeability by 1.18, 5.8, and 9.2 times, respectively (Figures 5C,D). Furthermore, PGE1 prolonged bleeding time in a concentration-independent manner. At doses of 35.4, 177, and 354 μg/kg, blood loss increased by 2.7-, 6.2-, and 3.4-fold, respectively, compared with the control (same volume of saline) (Figure 5E). PGE1 also suppressed acute pain. The mean licking time was 0.25 s in the mice administered with 10 nM PGE1 and 6.25 s in the control mice (Figure 5F). The analgesic effects of PGE1 may inhibit inflammation in order to stay undetected by the host. These processes balance pro-inflammatory factors, anti-inflammatory factors, and inhibiting platelet activation (20).

## Discussion

Increasing vascular permeability in the host and accelerating blood flow to the feeding site are indispensable processes for leeches to successfully obtain a blood meal. Histamine is found in leech saliva and is reported to increase vascular permeability (22). However, it is not the specific vasodilator in leech saliva since histamine is known to induce pain, which would antagonize the interests of the leech.

Interestingly, the potent platelet aggregation-inhibiting component of the leech was identified as PEG1, which was the major active component in the salivary gland. To the best of our knowledge, this is the first discovery of PEG1 in an invertebrate. The tissue distribution of PGE1 in the leech was mainly concentrated in the head, where the salivary gland and blood-sucking apparatus are located. Previous study has shown that PGE1 is a multi-functional molecule in mammals, including humans (23).

In our experiments, PGE1 inhibited platelet aggregation in a dose-dependent manner with an IC_50_ of 21.81 ± 2.24 nM, in accordance with other reports (24). Platelets play an important role in hemostasis. The involvement of PGE1 in leech saliva to prevent hemostasis likely ensures blood flow to the feeding site. In addition, PGE1 exhibited a vasodilatory effect (Figure 5). Clinical research has shown that systemic administration of PGE1 rapidly restores retinal blood flow through its vasodilatory effects (25). PGE1 in the salivary gland of leeches may have the same function to facilitate blood flow to the feeding site. At low concentrations, PGE1 also shows anti-inflammatory and analgesic effects (26). Several studies have confirmed that PGE1 decreases adhesion of monocytes by reducing the expression of vascular cell adhesion molecule-1 (27). It also inhibits NF-κB activation and reactive oxygen species production, resulting in the suppression of vascular inflammation (28, 29). PGE1 has been shown to alleviate neurological pain and dysfunction in diabetic rats at the entrapment site (30–32). An important factor to guarantee successful blood meals for leeches is to attach at the feeding site long enough to draw blood, but to escape the host immune response and prevent nociception. Thus, the leech takes advantage of the multi-functional molecule, PGE1, to adapt to its hematophagic life.

As PGE1 has never been reported in invertebrates, we are doubtful about the source of PGE1 in forest leeches. There are three possible sources of PGE1, i.e., synthesis by the leech itself, synthesis by symbiotic bacteria, and enrichment from host blood. We examined symbiotic bacterial culture but did not detect PGE1 (data not shown). However, we did find that the forest leech has prostaglandin synthesis-related genes through transcriptome analysis. Due to the difficultly in feeding forest leeches, we were unable to verify whether it is enriched from host blood.

Regardless of the source of PGE1 in leeches, this study confirmed that PGE1 does exist in invertebrates. This discovery expands the natural sources of PGE1. At the same time, the biological function of PGE1 showed it to be an important substance for leeches regarding their adaptation to a blood sucking lifestyle. This discovery indicates that the material basis of animal adaptive survival is not only polypeptides and proteins. We should also pay attention to small molecule compounds in animals in future research.

## Conclusions

In this study, using activity-guided analysis, PGE1 was found to be an efficient molecular tool in forest leech blood sucking. The structure of PGE1 was analyzed by NMR and HRESIMS. To determine the functions of PGE1 related to blood sucking, we studied how PGE1 affects platelet aggregation, skin vascular permeability, bleeding time, and pain. This discovery expands the natural sources of PGE1. The biological function of PGE1 showed that it is an important substance for blood sucking in leeches. This study also provides a new concept for the treatment and prevention of forest leech bite.

## Data Availability Statement

The original contributions presented in the study are included in the article/supplementary material, further inquiries can be directed to the corresponding author/s.

## Ethics Statement

The animal study was reviewed and approved by Animal Care and Use Committee at the Kunming Institute of Zoology, Chinese Academy of Sciences (SYDW-2013018). Written informed consent was obtained from the owners for the participation of their animals in this study.

## Author Contributions

CL and XL conceived and designed study. FZ, MZ, XY, and FW performed research. GW and RO analyzed data. JL, CY, RZ, and XF contributed new methods and models. RL, CL, and XL wrote the paper. All authors contributed to the article and approved the submitted version.

## Conflict of Interest

The authors declare that the research was conducted in the absence of any commercial or financial relationships that could be construed as a potential conflict of interest.
